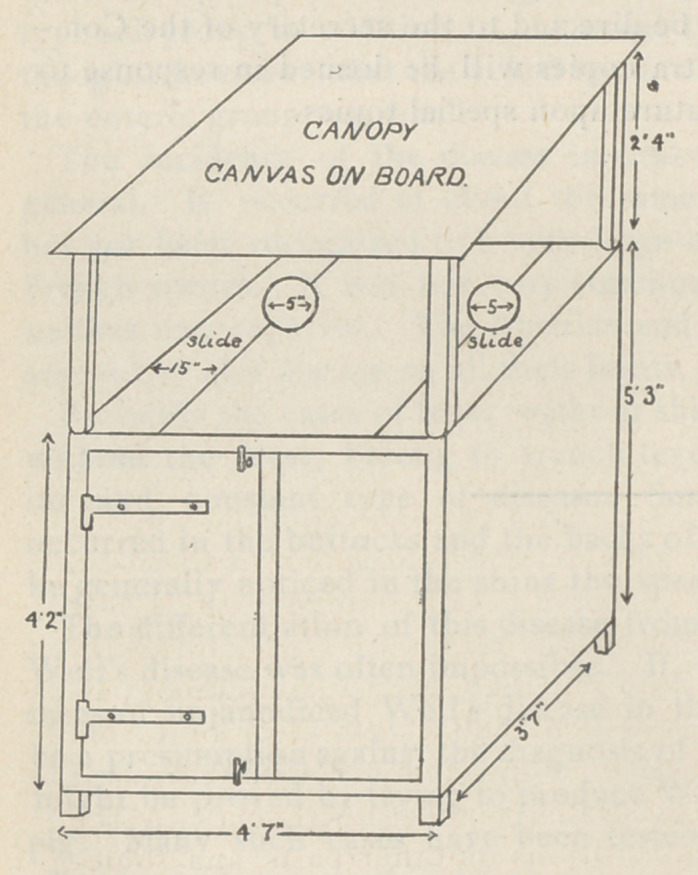# Treatment of Scabies by Sulphur Vapor

**Published:** 1918-01

**Authors:** 


### SCABIES

***Treatment of Scabies by Sulphur Vapor.*** By Major John Bruce, R. A. M. C. (T.), and Capt. Stanley Hodgson, R. A. M. C. Abs. from the *Brit. Med. Jour.*, Aug. 5, 1916.

Since treatment by sulphur inunction is slow, requiring from 1 to 2 weeks, the authors tried sulphur dioxide gas for twelve months and found it thoroughly satisfactory.

They improvised a cabinet of the "home Turkish bath" type large enough to accommodate two patients at a time. The treatment is given out of doors. The patient is first given a hot bath and allowed to soak for five minutes, with the usual scrubbing with soap to open the burrows. He is then transferred to the cabinet, and placed on the seat with his head protruding through the aperture in the roof of sliding boards. A wet towel is applied around the neck to prevent the escape of the fumes. A large Jeyes' sulphur candle is preferable, as a quick-burning candle with a large wick is likely to prove irritating. The bath lasts fifty minutes, and during this time an attendant should be present in case there should be fainting or too free an escape of fumes. In the meantime all the clothing should be treated, either by means of a Thresh or steam disinfector, or by being placed in the chamber with the patient. It is especially necessary to treat the bedding. No antiseptics should be used with the treatment as they cause irritation. In cold weather it is best to heat the box first with an oil stove.

The treatment gives immediate and lasting relief from itching. It has cured all but about 2 o/o of the cases, and the failure with these, the authors believe, was due to insufficient treatment of the kit. Men may return to their unit the same day, and resume duty the next. One box will serve about 20 cases a day.

## Figures and Tables

**Figure f1:**